# Supplementary figures and images for: Gene redundancy and gene compensation of insulin-like peptides in the oocyte development of bean beetle
Source: PLoS One. 2024 May 7;19(5):e0302992. doi: 10.1371/journal.pone.0302992 (PMC11075890; doi:10.1371/journal.pone.0302992)

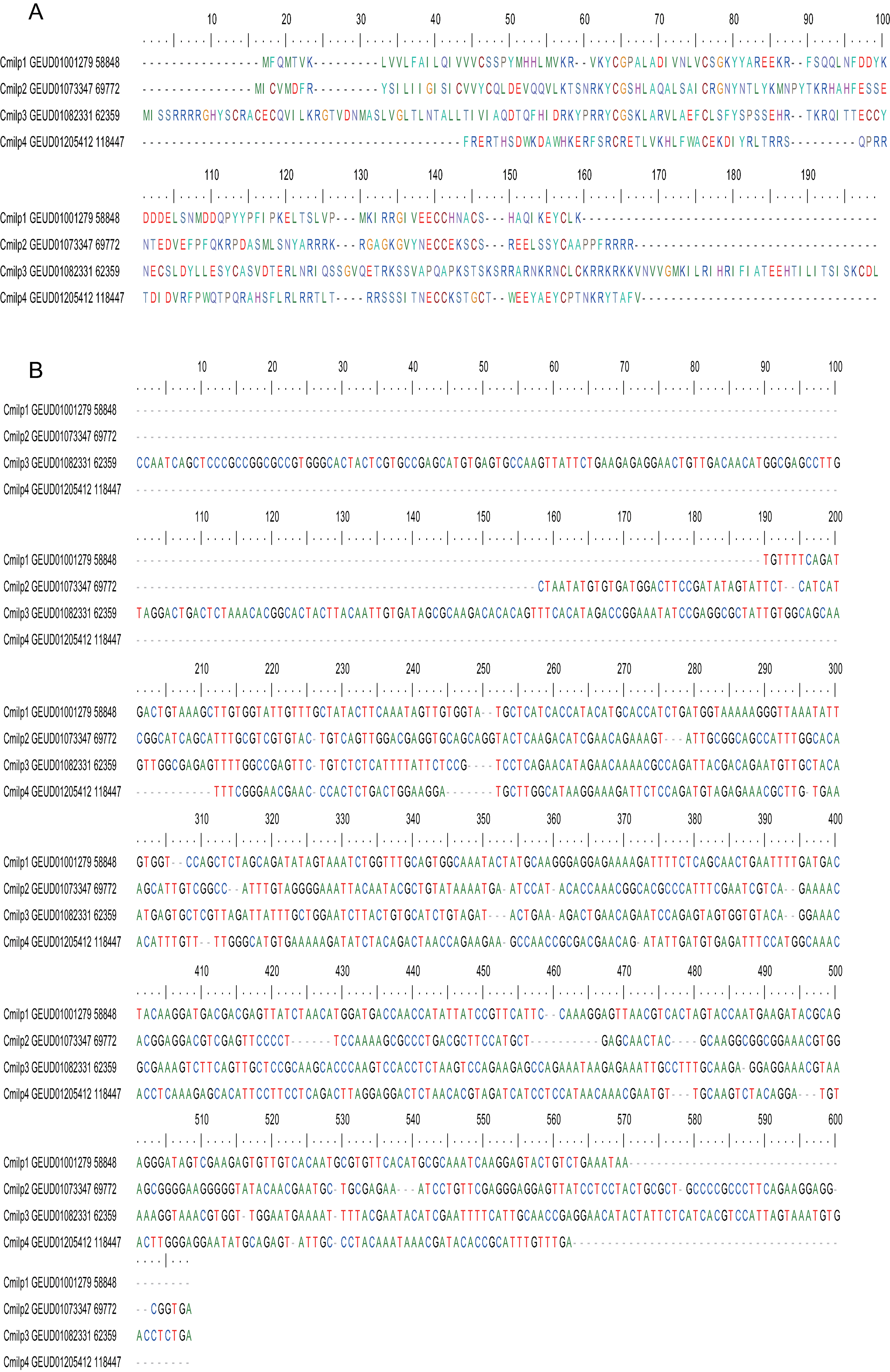

Supplement: S1 Fig — (A) Protein sequence, (B) nucleotide sequence. (TIF) [file pone.0302992.s001.tif]

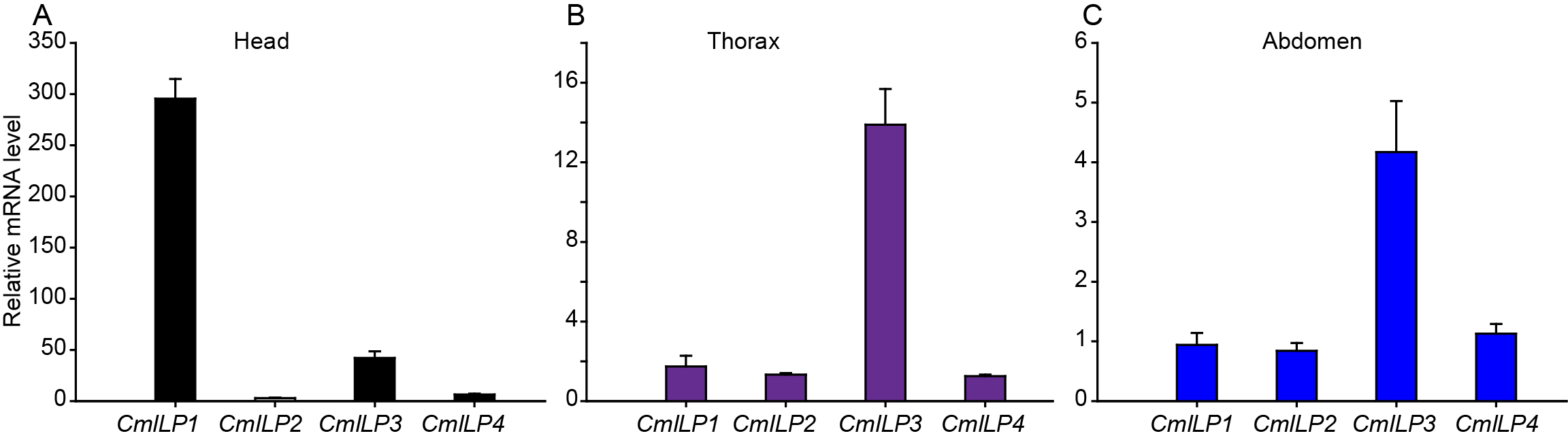

Supplement: S2 Fig — Expression of insulin-like peptides in head (A), thorax (B), and abdomen (C). (TIF) [file pone.0302992.s002.tif]
