# Supplementary material for: Gene redundancy and gene compensation of insulin-like peptides in the oocyte development of bean beetle
Source: PLoS One. 2024 May 7;19(5):e0302992. doi: 10.1371/journal.pone.0302992 (PMC11075890; doi:10.1371/journal.pone.0302992)
Supplement: S1 Table — (DOCX) [file pone.0302992.s003.docx]

**Table S1.** Primers used for RT-qPCR and RNAi.

| Primer name | Gene ID | Location | Exon | Sequence (5' to 3') |
| --- | --- | --- | --- | --- |
| q-rp49 F | GEUD01120135.1 | 197-216 | 🗸 | GCAACTGGCGTAAACCTAAA |
| q-rp49 R |  | 319-338 | 🗸 | TGTACTAGCACCTTCCTGAA |
| q-CmILP1 F | GEUD01205412.1 | 160-179 | 🗸 | TGAAACAGCGTTACAGTCCG |
| q-CmILP1 R |  | 320-339 | 🗸 | GGTTGGCTTCTTCTGGTTAG |
| q-CmILP2 F | GEUD01073347.1 | 357-376 | 🗸 | AGCAGGTACTCAAGACATCG |
| q-CmILP2 R |  | 536-555 | 🗸 | CTCAGCATGGAAGCGTCAGG |
| q-CmILP3 F | GEUD01082331.1 | 255-274 | 🗸 | CTGACTCTAAACACGGCACT |
| q-CmILP3 R |  | 361-380- | 🗸 | GAGACAGAACTCGGCCAAAA |
| q-CmILP4 F | GEUD01001279.1 | 102-118 | 🗸 | CATCACCATACATGCACCAT |
| q-CmILP4 R |  | 245-264 | 🗸 | GATAACTCGTCGTCATCCTT |
| RNAi-GFP F | MT612434.1 | 2322-2340 | 🗸 | CACAAGTTCAGCGTGTCCG |
| RNAi-GFP R |  | 2723-2741 | 🗸 | GTTCACCTTGATGCCGTTC |
| RNAi-CmILP1 F | GEUD01205412.1 | 204-223 | 🗸 | TTCGGGAACGAACCCACTCT |
| RNAi-CmILP1 R |  | 464-483 | 🗸 | TCCTCCCAAGTACATCCTGT |
| RNAi-CmILP2 F | GEUD01073347.1 | 307-326 | 🗸 | CATCATCGGCATCAGCATTT |
| RNAi-CmILP2 R |  | 458-477 | 🗸 | TGCCGTTTGGTGTATGGATT |
| RNAi-CmILP3 F | GEUD01082331.1 | 413-432 | 🗸 | AAAACGCCAGATTACGACAG |
| RNAi-CmILP3 R |  | 574-593 | 🗸 | TCTTCTGGACTTAGAGGTGG |
| RNAi-CmILP4 F | GEUD01001279.1 | 186-205 | 🗸 | GCAAATACTATGCAAGGGAG |
| RNAi-CmILP4 R |  | 361-380 | 🗸 | CATGTGAACACGCATTGTGA |
| T7-RNAi-GFP F |  |  |  | TAATACGACTCACTATAGGCACAAGTTCAGCGTGTCCG |
| T7-RNAi-GFP R |  |  |  | TAATACGACTCACTATAGGGTTCACCTTGATGCCGTTC |
| T7-RNAi-CmILP1 F |  |  |  | TAATACGACTCACTATAGGTTCGGGAACGAACCCACTC |
| T7-RNAi-CmILP1 R |  |  |  | TAATACGACTCACTATAGGTCCTCCCAAGTACATCCTGT |
| T7-RNAi-CmILP2 F |  |  |  | TAATACGACTCACTATAGGCATCATCGGCATCAGCATTT |
| T7-RNAi-CmILP2 R |  |  |  | TAATACGACTCACTATAGGTGCCGTTTGGTGTATGGATT |
| T7-RNAi-CmILP3 F |  |  |  | TAATACGACTCACTATAGGAAAACGCCAGATTACGACAG |
| T7-RNAi-CmILP3 R |  |  |  | TAATACGACTCACTATAGGTCTTCTGGACTTAGAGGTGG |
| T7-RNAi-CmILP4 F |  |  |  | TAATACGACTCACTATAGGGCAAATACTATGCAAGGGAG |
| T7-RNAi-CmILP4 R |  |  |  | TAATACGACTCACTATAGGCATGTGAACACGCATTGTGA |
