# Supplementary material for: Gene redundancy and gene compensation of insulin-like peptides in the oocyte development of bean beetle
Source: PLoS One. 2024 May 7;19(5):e0302992. doi: 10.1371/journal.pone.0302992 (PMC11075890; doi:10.1371/journal.pone.0302992)
Supplement: S2 Table — (DOCX) [file pone.0302992.s004.docx]

**Table S2.** Sequence identity and similarity among ILPs

| Cmilp1 |  | Tcilp1 | Tcilp2 | Tcilp3 | Tcilp4 | Dmilp1 |
| --- | --- | --- | --- | --- | --- | --- |
|  | I |  | 26% | 27% |  | **34%** |
|  | P |  | 42% | 36% |  | **48%** |
|  | G |  | 7% | 44% |  | **17%** |
| Cmilp2 | I |  | **35% (47/133)** | 35% (13/37) | 38% (19/49) |  |
|  | P |  | **58% (78/133)** | 54% (20/37) | 53% (26/49) |  |
|  | G |  | **8%** | 18% | 2% |  |
| Cmilp3 | I |  |  | **33% (43/130)** |  |  |
|  | P |  |  | **51% (67/130)** |  |  |
|  | G |  |  | **11%** |  |  |
| Cmilp4 | I |  |  |  | **64%** |  |
|  | P |  |  |  | **81%** |  |
|  | G |  |  |  | **4%** |  |

I, Identity

P, Positives

G, Gap
