# Supplementary material for: Gene redundancy and gene compensation of insulin-like peptides in the oocyte development of bean beetle
Source: PLoS One. 2024 May 7;19(5):e0302992. doi: 10.1371/journal.pone.0302992 (PMC11075890; doi:10.1371/journal.pone.0302992)
Supplement: Attachment — Submitted filename: response to reviewers.docx [file pone.0302992.s006.docx]

**Rebuttal letter to the editors and reviewers (original comments from the editors and reviewers are in blue, and our responses are in black)**

Comment 1: Please ensure that your manuscript meets PLOS ONE's style requirements, including those for file naming. The PLOS ONE style templates can be found at https://journals.plos.org/plosone/s/file?id=wjVg/PLOSOne_formatting_sample_main_body.pdf and <https://journals.plos.org/plosone/s/file?id=ba62/PLOSOne_formatting_sample_title_authors_affiliations.pdf>

Response: Thank you for the comment! Our manuscript meets the style requirements of PLOS ONE.

Comment 2: We suggest you thoroughly copyedit your manuscript for language usage, spelling, and grammar. If you do not know anyone who can help you do this, you may wish to consider employing a professional scientific editing service.

Whilst you may use any professional scientific editing service of your choice, PLOS has partnered with both American Journal Experts (AJE) and Editage to provide discounted services to PLOS authors. Both organizations have experience helping authors meet PLOS guidelines and can provide language editing, translation, manuscript formatting, and figure formatting to ensure your manuscript meets our submission guidelines. To take advantage of our partnership with AJE, visit the AJE website (http://learn.aje.com/plos/) for a 15% discount off AJE services. To take advantage of our partnership with Editage, visit the Editage website (www.editage.com) and enter referral code PLOSEDIT for a 15% discount off Editage services.  If the PLOS editorial team finds any language issues in text that either AJE or Editage has edited, the service provider will re-edit the text for free.

Upon resubmission, please provide the following:

● The name of the colleague or the details of the professional service that edited your manuscript

● A copy of your manuscript showing your changes by either highlighting them or using track changes (uploaded as a *supporting information* file)

● A clean copy of the edited manuscript (uploaded as the new *manuscript* file)

 Response: Thank you for the comment. English grammar has been checked by a native English speaker.

Comment 3: Thank you for stating the following financial disclosure:

   "This research was funded by National Natural Science Foundation of China (grant number 32060124), Guizhou Normal University (grant number Qianshixinmiao [2021] A11), Guizhou Provincial Science and Technology Foundation (grant number Qiankehejichu[2020]1Y080), the Joint Fund of the National Natural Science Foundation of China and the Karst Science Research Center of Guizhou Province (U1812401), Provincial Program on Platform and Talent Development of the Department of Science and Technology of Guizhou China (grant number [2019]5617, [2019]5655 and [2017]5726-21)."

Please state what role the funders took in the study.  If the funders had no role, please state: "The funders had no role in study design, data collection and analysis, decision to publish, or preparation of the manuscript."

If this statement is not correct you must amend it as needed.

Please include this amended Role of Funder statement in your cover letter; we will change the online submission form on your behalf.

Response: The funders had no role in study design, data collection and analysis, decision to publish, or preparation of the manuscript. The sentence was added in cover letter.

Comment 4: Please include captions for your Supporting Information files at the end of your manuscript, and update any in-text citations to match accordingly. Please see our Supporting Information guidelines for more information: http://journals.plos.org/plosone/s/supporting-information.

Response: Captions for your Supporting Information files have been added at the end of the manuscript.

Fig S1. Sequence alignment of *CmILPs*. (A) Protein sequence, (B) nucleotide sequence.

Table S1. Primers for RT-qPCR and RNAi.

Table S2. Sequence identity and similarity among ILPs.

Table S3. Raw data.

Comment 5: Additional Editor Comments:

In addition to the Reviewer comments (appended at the bottom of this email), I have provided a number of my own comments (see below).

1) I highly encourage you to seek the input from a scientific editing service or a native English-speaking colleague with molecular experience.

2) For the Introduction, shift the focus of the first paragraph away from mouse & zebrafish to insects

3) Results - if data is presented in a figure, then there is no need to also include it in the text. As currently written, the readability of the Results is hampered by the numerous values listed. Consider highlighting the main points in the Figures rather than listing every percentage value.

4) Provide a sequence alignment figure showing the lack of identity mentioned in line 205.

5) Provide significantly more methodological details. As currently written, they are insufficient to allow for replication.

- provide accession/bioproject numbers for the transcriptome and genome searched. Also, provide accession numbers for the sequences used as queries.

- In the phylogenetic analysis, provide information/details on bootstrap support, the model/method used, how gaps/missing data were treated, and indicate if full-length sequences were used. Was the signal peptide removed prior to analysis? Also, provide accession numbers for the sequences used to construct the phylogenetic tree.

- Was gDNA removed prior to cDNA synthesis?

- Is there a reference indicating the suitability of using rpl49 as a reference gene?

- Were the PCR product sequences validated?

- What were the qRT-PCR conditions?

- How was the dsRNA synthesis template generated?

- How were dsRNAs injected? was a microinjection system used? where was the injection site? what types of needles were used?

- By "silencing efficiency" do the authors mean confirmation of transcript knockdown? provide PCR conditions

- How were terminal oocyte measurements made?

6) In the Results, indicate how the ILPs were identified (ie based on sequence homology with query sequences or via previous annotation) and indicate what the sequence identity/similarity was with the other ILPs.

Response:

1) English grammar of the manuscript has been checked by scientific editing service.
2) We shifted the focus of the first paragraph away from mouse & zebrafish to insects.
3) Thank you for the comment! We described the main points of the Figures.
4) A sequence alignment figure was added (Fig S1).
5) Bioproject numbers for the transcriptome (PRJNA309272) and genome (PRJEB62873) have been added. Accession numbers for the sequences were listed in Figure 2A and Table S1.

The full length of protein sequences of the ILPs of the five species mentioned above were used to construct a phylogenetic tree using Neighbor-Joining algorithms in MEGA 11.0. Bootstrapped values were calculated with 1000 replications. Substitution model was Poisson model. Pairwise deletion was used for gaps/missing data treatment.

Genomic DNA (gDNA) was removed prior to cDNA synthesis.

Ribosome protein 49 (rp49) is usually used as reference gene (Chen Q, He J, Ma C, Yu D, Kang L. Syntaxin 1A modulates the sexual maturity rate and progeny egg size related to phase changes in locusts. Insect Biochemistry and Molecular Biology. 2015;56:1-8. doi: <https://doi.org/10.1016/j.ibmb.2014.11.001>; Wei J, Shao W, Cao M, Ge J, Yang P, Chen L, et al. Phenylacetonitrile in locusts facilitates an antipredator defense by acting as an olfactory aposematic signal and cyanide precursor. Science Advances. 2019;5(1):eaav5495. doi: doi:10.1126/sciadv.aav5495.)

PCR product sequences were validated by Sanger sequencing.

The qRT-PCR conditions were initial denature at 95℃ for 2 min, 40 cycles at 95℃ for 15 s, annealing temperature 58℃ for 20 s, extension temperature 72℃ for 20 s, and final extension 72℃ for 10 min. melting curve analysis (58-95℃) was applied to all reactions to ensure the specificity of the PCR product.

Four *ILPs* were amplified using cDNA with gene-specific primers (Table S1), then cloned into pGEM-T Easy vector (Promega, Madison, USA). dsRNA synthesis templates were generated using the vector containing gene-specific sequence with gene-specific primers containing the T7 promoter sequence at the 5＇end of forward primer or reverse primer.

At the pupal stage, approximately one µg of corresponding dsRNA was injected into each beetle through the abdomen using a nanoliter injector 2000 (World Precision Instruments) with micro glass needle.

Transcript knockdown was used to determine silencing efficiency. The PCR conditions mentioned above.

The ovarioles were separated from each other and fixed in 4% paraformaldehyde overnight. The terminal oocytes were captured with a stereomicroscope (SZX7 Olympus, Japan), then the length of terminal oocytes was measured with software cellSens (Olympus, Japan).

6) In the results, the ILPs were identified based on sequence homology with query sequences. The sequence identity/similarity between *CmILPs* and *TcILPs* was listed in Table S2.

Comment 6: Reviewer #1: The manuscript by Li and co-workers reports the occurrence of four ILPs in the bean beetle, their expression in different culture conditions and parts of the body. In addition, they report the effects of different RNAi treatments on oocyte length and ILP mRNA levels. The work is generally well executed and the results are interesting. However, they are quite scarce. Only the expression of ILPs is quantified and no others analyses or experiments are done beyond measuring the length of the oocytes.

Response: terminal oocyte length and egg size are usually used to measure insects’ reproduction. In previous study, we found there was no difference in egg size between high density individuals and low density individuals (Chen et al., 2019, Annales Zoologici Fennici), as a result, we just measured terminal oocyte length in the study.

Comment 7: In addition, some other questions must be considered.

-Data availability: According to the PLoS One data availability policies, the values behind the means, standard deviations and other measures must be available in the manuscript or in a repository. This provision is not met in the case of this work.

Response: All raw data which used for analysis were listed in Table S3.

Comment 8: -The manuscript needs a revision of the English language of the text.

Response: The English language has been checked by a native English speaker.

Comment 9: -In Material and methods, it is stated that qPCR calculations are performed with the 2-ΔΔCt method, but in the case of Fig.2 B-D it seems that 2-ΔCt instead of 2-ΔΔCt has been used. Is this right?

-Material and methods, statistical analysis: Levene test does not analyze the normality of data. If you want to analyze normality you must use an ad hoc test as, for example, Shapiro-Wilk.

Response: Relative gene expression values were calculated using the 2^-△△Ct^ method. In Fig.2 B-D, the expression levels of other samples were normalized to the first sample of Abdomen (raw data were listed in Table S3).

Normality and equality of data variances were checked using the ShapiroeWilk test.

Comment 10: -The results are overexplained and reading the text becomes not only tedious, but even complicated.

-Line 73. Please change “maculate” by “maculatus”.

-Results sexual maturity rate. Although it is mentioned in the Material and Methods section, please indicate also here at what time did you measure the terminal oocyte length for both high- and low-density animals.

-I think it is necessary for the authors to include the sequences of the bean beetle ILPs in NCBI or in another public repository, at least from the time of publication, but I have not been able to find any Accession Number or reference in this regard.

Response: We tried our best to make the manuscript more concise.

“maculate” was replaced by “maculatus”.

The information of time was added in result (Within 12 hours after the emergence of the adult, females were soaked in 4% paraformaldehyde solution for 72 hours. Then the ovarioles were separated from each other for terminal oocyte length measurement).

Accession Number of all sequences were added in Figure 2A and Table S1.

Comment 11: -Discussion: Phylogenetic tree. The number of each ILP usually does not correspond to that determined by the orthology with other ILPs, since it is very difficult to find the orthologous peptides (see, for example, Antonova et al 2012. Insulin-Like Peptides: Structure, Signaling, and Function. In: Gilbert, L. I. (Ed.), Insect Endocrinology. Elsevier, pp. 63–92). In the case of Callosobruchus, I understand that the ILPs have been named because of their similarity to those of Tribolium, a relatively close species. However, that will not be the case with the other species included in the phylogenetic tree, so it is normal that the numbers do not coincide. I consider the phylogenetic tree to be of limited interest. -There is a clear example of compensation at the level of ILP expression and not only at reproduction or development level in the case of the cockroach Blatella germanica (Castro-Arnau et al. 2019. J. Insect Physiol. 114, 57-67).

-Discussion, lines 361-362. Reference 10 refers to Nilaparvata and not to Tribolium.

-Discussion, lines 367-369. Please, indicate the organism to which this statement refers.

-How do you explain that dsCmILP3 alone and dsCmILP4 alone produce 86.80% and 88.17% reduction of brain CmILP2 mRNA levels, respectively, but dsCmILP3&ILP4 do not modify them?

Response: ILPs of *Callosobruchus maculatus* were named based on sequence similarity with ILPs of *Tribolium castaneum*, a relatively close species. Owing to ILPs exhibit high degree structural conservation rather than amino acid sequence conservation [5], the phylogenetic tree was constructed with amino acid sequence, as a result, it was normal that the numbers did not coincide among different species.

The reference (Castro-Arnau et al. 2019. J. Insect Physiol. 114, 57-67) was cited in our manuscript.

The reference (lines 361-362. Reference 10 refers to Nilaparvata and not to Tribolium) has been corrected.

Zebrafish (Discussion, lines 367-369. Please, indicate the organism to which this statement refers) was added.

In fact, we cannot answer the question (How do you explain that dsCmILP3 alone and dsCmILP4 alone produce 86.80% and 88.17% reduction of brain CmILP2 mRNA levels, respectively, but dsCmILP3&ILP4 do not modify them?). Injection of dsCmILP3 alone and dsCmILP4 alone produced 86.80% and 88.17% reduction of brain CmILP2 mRNA levels, respectively, however, injection of dsCmILP3&ILP4 had no significant change on brain CmILP2 mRNA levels. The mechanism needs further study.

Comment 12: -First paragraph of the Introduction and last paragraph of the discussion. In the dsRNA treatments, the RNA is degraded through the RNAi pathway using the RNase activity of Argonaute in the RNA-induced silencing complex (RISC), and not using the nonsense-mediated decay (NMD) pathway. Then, I don’t think the information showed in those paragraphs is really relevant.

Response: We revised the first paragraph. We focused on gene redundant and gene compensation in insects.

Comment 13: -Figure 2A. What do the colors mean?

Response: We used different colors to discriminate different ILPs.

Comment 14: Reviewer #2: In the manuscript by Li et al., the authors identified the expression pattern of insulin like peptides in the bean beetle, and examined the function of these ILPs in oocyte development by RNAi. The experiments are well designed, and the results are solid and support the main conclusions. The manuscript is presented in an intelligible fashion and well written. I only have a few concerns that the authors should address:

1. The authors stated that the female sexual maturity rate could be indicated by the length of terminal oocytes. I feel that this should be better explained in the Introduction. Why and how does long terminal oocyte represent fast sexual maturity rate? In my opinion, sexual maturation means the females are ready for mating with males and laying eggs. A group of female beetles have longer terminal oocytes at 12hrs after emergence does not necessarily mean that they will mature faster. If no further experimental evidence could be provided, I would suggest to describe the results as ILPs are involved in oocyte development or terminal oocyte maturation.

Response: Thank you for the suggestion. We used terminal oocyte maturation in the manuscript. We added the importance of terminal oocyte maturation in introduction.

Comment 15: 2. The first paragraph of the introduction focuses on "Gene redundancy", which seems not to be important and necessary. The examples in this paragraph are not very relevant with ILPs nor insects. Gene redundancy and compensation have been studied in Drosophila ILPs, as the authors described in Discussion.

Response: The first paragraph focused on gene redundancy and compensation in insects.

Comment16: 3. The writtings could be further improved.

Response: We revised the manuscript according to comments.

Comment17: While revising your submission, please upload your figure files to the Preflight Analysis and Conversion Engine (PACE) digital diagnostic tool, <https://pacev2.apexcovantage.com/>. PACE helps ensure that figures meet PLOS requirements. To use PACE, you must first register as a user. Registration is free. Then, login and navigate to the UPLOAD tab, where you will find detailed instructions on how to use the tool. If you encounter any issues or have any questions when using PACE, please email PLOS at [figures@plos.org](mailto:figures@plos.org). Please note that Supporting Information files do not need this step.

Response: All figures were checked by PACE.
