# Supplementary material for: Gene redundancy and gene compensation of insulin-like peptides in the oocyte development of bean beetle
Source: PLoS One. 2024 May 7;19(5):e0302992. doi: 10.1371/journal.pone.0302992 (PMC11075890; doi:10.1371/journal.pone.0302992)
Supplement: Attachment — Submitted filename: response to reviewers.docx [file pone.0302992.s007.docx]

**Rebuttal letter to the editors and reviewers (original comments from the editors and reviewers are in blue, and our responses are in black)**

Comment 1: Reviewer 1 had a number of additional suggestions for you to consider that would further enhance the overall clarity of the manuscript. In addition, I would recommend that further copy editing via a native English speaking colleague or editing service be considered. Although many grammatical problems in the original manuscript were addressed, some issues still persist.

Response: Thank you for the comment! English grammar has been checked by a native English speaker again.

Comment 2: Reviewer #1: The new version of the manuscript has incorporated most of the changes suggested by this reviewer. However, I still have some comments regarding the new version. I indicate these comments below.

-There are some parts of the first paragraph of the introduction that are repeated in the discussion. I think the entire first paragraph of the introduction should be removed and all information passed through, just once, into the discussion.

Response: Thank you for the suggestion! The entire first paragraph of the introduction was removed.

Comment 3: -Given the bootstrap numbers observed in the phylogenetic tree, is it possible that the Bootstrapped values were calculated with 100 replications instead of 1000 replications as stated on line 110?

Response: The information (bootstrap replicate support percentages are shown next to the branches) was added in figure legends (line 451).

Comment 4: -Please, indicate how did you eliminate genomic DNA.

Response: Genomic DNA (gDNA) was removed with DNase in FastKing gDNA Dispelling RT SuperMix (Tiangen) (line 108).

Comment 5: -Perhaps due to the new tree that positions the ILPs in places different from those of the original manuscript, there must have been an exchange of names between CmILP1 and CmILP4. Make sure this nomenclature change has also been corrected in the raw data. I think that, at least for some values in Fig.2, that change has not been made.

Response: Thank you for the comment! We corrected the raw data.

Comment 6: - I would like to see, even if it is in supplementary material, a figure that directly compares the expressions of the different ILPs in the different tissues (head, thorax and abdomen). That is: a graph with the four ILPs in the head, another in the thorax and another in the abdomen. I think this result is interesting to understand to what extent the increase in ILP3 expression in the abdomen of dsILP1 and dsILP2 can compensate for the reduction in ILP1 and ILP2, respectively. The fact that in Figure 2 B-E the expression in the abdomen for each of the ILPs is used as a “calibrator” makes the comparison of the ILPs in each tissue not direct because it depends on the respective expressions in the abdomen.

Response: Fig S2 was added (line 200).

Comment 7: -The text in the section “Function of ILPs in terminal oocyte maturity rate” could be greatly reduced considering that the figures are already there.

Response: Thank you for the comment! The corresponding description was greatly reduced.

Comment 8: -How many different animals were used for terminal oocyte length measurements and how many oocytes were measured from each animal? Please, indicate this somewhere in the manuscript.

Response: Ten terminal oocytes from each female were measured, then the ten terminal oocytes’ lengths were calculated as an average value (line 88). The number of females which were used for the measurement of terminal oocyte length would be described in the text (Fig 5D, E, n= 12 and 10 for dsGFP and dsCmILP1&ILP3, respectively; *t*-test *P* < 0.001).

Comment 9: Reviewer #2: The authors have addresed my concerns as well as those risen by another reviewer and the editor. I have no further questions.

Response: Thank you for your time and dedication in reviewing our manuscript.
